# Unlocking the Sporicidal Potential of Ethanol: Induced Sporicidal Activity of Ethanol against *Clostridium difficile* and *Bacillus* Spores under Altered Physical and Chemical Conditions

**DOI:** 10.1371/journal.pone.0132805

**Published:** 2015-07-15

**Authors:** Michelle M. Nerandzic, Venkata C. K. Sunkesula, Thriveen Sankar C., Peter Setlow, Curtis J. Donskey

**Affiliations:** 1 Research Service, Cleveland Veterans Affairs Medical Center, Cleveland, Ohio, United States of America; 2 Case Western Reserve University School of Medicine, Cleveland, Ohio, United States of America; 3 Department of Molecular Biology and Biophysics, University of Connecticut Health Center, Farmington, Connecticut, United States of America; 4 Geriatric Research, Education and Clinical Center, Cleveland Veterans Affairs Medical Center, Cleveland, Ohio, United States of America; Institute Pasteur, FRANCE

## Abstract

**Background:**

Due to their efficacy and convenience, alcohol-based hand sanitizers have been widely adopted as the primary method of hand hygiene in healthcare settings. However, alcohols lack activity against bacterial spores produced by pathogens such as *Clostridium difficile* and *Bacillus anthracis*. We hypothesized that sporicidal activity could be induced in alcohols through alteration of physical or chemical conditions that have been shown to degrade or allow penetration of spore coats.

**Principal Findings:**

Acidification, alkalinization, and heating of ethanol induced rapid sporicidal activity against *C*. *difficile*, and to a lesser extent *Bacillus thuringiensis* and *Bacillus subtilis*. The sporicidal activity of acidified ethanol was enhanced by increasing ionic strength and mild elevations in temperature. On skin, sporicidal ethanol formulations were as effective as soap and water hand washing in reducing levels of *C*. *difficile* spores.

**Conclusions:**

These findings demonstrate that novel ethanol-based sporicidal hand hygiene formulations can be developed through alteration of physical and chemical conditions.

## Introduction

Effective hand hygiene is essential to prevent transmission of healthcare-associated pathogens [1,2]. Due to their efficacy and convenience, alcohol-based hand sanitizers, typically 60–80% ethanol or isopropanol, have been widely adopted as the primary method of hand hygiene in healthcare settings [3–6]. The antimicrobial activity of alcohols is attributed to their ability to denature proteins, resulting in potent germicidal activity against vegetative bacteria and many fungi and enveloped viruses [7]. However, alcohols lack activity against bacterial spores and use of alcohol hand sanitizer does not reduce levels of spores on hands [8–10]. This gap in the spectrum of activity of alcohols is of critical importance because the spore-forming anaerobe *Clostridium difficile* is a major pathogen in healthcare facilities and in the community [11–13]. Recent evidence that asymptomatic carriers of *C*. *difficile* may be an important source of transmission is particularly concerning [14]. Asymptomatic carriers outnumber symptomatic patients and shed spores onto skin and environmental sites touched by healthcare workers, but they remain unidentified and alcohol is used for hand hygiene [14,15]. Soap and water handwashing is currently the gold-standard for removing spores from hands and is indicated when contact with spores is suspected or likely [1]. However, soap and water handwashing is only moderately effective and use of handwashing in place of instant alcohol-based hand sanitizers is not feasible for widespread adoption [8–10]. Thus, there is a need for the development of hand hygiene strategies that are both efficient and effective in reducing the burden of spores on hands.

Modest success in reducing levels of *C*. *difficile* spores on hands has been achieved using alternatives to alcohol hand sanitizers. In healthy subjects whose hands were inoculated with spores, hand washing with soap and water reduced spore recovery by ~1.5 to 2 log_10_ colony-forming units (CFU) through mechanical removal [8–10]. In patients with *C*. *difficile* infection or asymptomatic carriage, soap and water hand washing for 30 seconds significantly reduced the frequency of positive cultures and the burden of spores on hands, whereas alcohol hand sanitizer did not [16]. In healthy test subjects, a >2 log reduction in *C*. *difficile* or *Bacillus* spp. spores on hands was achieved by soaking for 1 minute in a sporicidal electrochemically generated hypochlorous acid solution [17]. These studies demonstrate that reductions in spores can be achieved on hands, but in a high-frequency application setting the hand hygiene methods used lack the convenience, efficiency, and skin compatibility of alcohol hand sanitizers.

Alcohol and other antiseptics may be ineffective against spores in part because they are unable to access sites of action in the spore core [18,19]. We hypothesized that use of alcohol or other antiseptics in combination with compounds or conditions that might degrade or allow penetration of spore coats could provide a novel approach for development of sporicidal hand hygiene products. There is some evidence that such approaches can be effective. For example, the skin antiseptic chlorhexidine does not kill spores under ambient conditions, but exhibits sporicidal activity against *Bacillus* spp. spores at elevated temperatures and in the presence of alcohol, elevated pH and ultra-sonication [20–22]. We have demonstrated that chlorhexidine exhibits sporicidal activity against *C*. *difficile* spores at elevated temperatures, with enhancement of activity at elevated pH or in the presence of 70% ethanol [23]. Gould et al. demonstrated that *Bacillus cereus* spores became susceptible to killing by lysozyme after exposure to reducing agents that damage the disulfide bonds in proteins [24]. Similarly, exposure to low pH has been shown to reduce the resistance of *Bacillus cereus* spores to sub-lethal heat treatments [25].

Here, we test the hypothesis that alteration of physical and chemical conditions (e.g., acid or alkaline pH and elevated temperature) can induce rapid sporicidal activity of alcohol against *C*. *difficile* and *Bacillus* spp. spores, both *in vitro* and on skin. This report demonstrates the potential for development of new ethanol-based sporicidal hand hygiene formulations through physical and chemical modifications.

## Materials and Methods

### Ethics Statement

*C*. *difficile* strains were isolated from patients at the Cleveland Veterans Affairs Medical Center. Clinical isolate strains were used for the *in vitro* and *ex vivo* porcine skin models of the study. The Institutional Review Board of the Cleveland Veterans Affairs Medical Center approved the study protocol (reference number: 11009-H10 approved 5/18/2011) for collection of all patient isolates and for hand hygiene studies. For the hand hygiene studies, a non-toxigenic *C*. *difficile* strain purchased from the American Type Culture Collection (ATCC) was used and participants provided verbal informed consent to participate. Verbal informed consent was obtained because the study was considered minimal risk. Participant consent was recorded in study documents. The Cleveland Veterans Affairs Institutional Review Board approved the consent procedure. The Institutional Review Board of the Cleveland Veterans Affairs Medical Center waived the need for written informed consent from the participants for the collection of *C*. *difficile* strains because the isolates were cultured from clinical samples with no collection of patient identifiers or interaction with subjects.

### Spore Strains and Growth Conditions

Two *C*. *difficile* strains cultured from patients with CDI in Cleveland and one strain purchased from the American Type Culture Collection (ATCC) were used. VA 17 is an epidemic (cdtB+) restriction endonuclease analysis (REA) BI strain and VA 11 is a non-epidemic (cdtB-) REA J strain; both isolates are toxigenic (tcdA+, tcdB+) strains. ATCC 43593 is a non-toxigenic (*tcdA*, *tcdB*-) strain from serogroup B. *C*. *difficile* cultures were incubated at 37°C for 48 hours in a Whitley MG1000 anaerobic workstation (Microbiology International, Frederick, MD) on pre-reduced cycloserine-cefoxitin-brucella agar containing 0·1% taurocholic acid and lysozyme 5 mg/L (CDBA) [26]. The Institutional Review Board of the Cleveland Veterans Affairs Medical Center approved the study protocol for collection of the patient isolates.

Two *Bacillus* species were used for *in vitro* studies. A well characterized strain of *Bacillus subtilis* (strain 168 containing plasmid pUB110 carrying a gene for kanamycin resistance) was donated by Peter Setlow (UConn Health Center, Farmington, Connecticut). A strain of *Bacillus thuringiensis* (ATCC 55173) was also assessed. *Bacillus* spores were cultured on trypticase soy agar (TSA) containing 5% sheep blood (Becton, Dickinson and Company, Franklin Lakes, New Jersey) under aerobic conditions at 37°C for 24 hours

### Preparation of Spores

*C*. *difficile* and *B*. *thuringiensis* spores were prepared as previously described [27]. In brief, pre-reduced brain-heart infusion (BHIS) plates were spread with 100 μl of a 24 hour *C*. *difficile* or *B*. *thuringiensis* suspension and incubated for one week in an anaerobic or aerobic incubator, respectively. Spores were harvested from the plates using sterile swabs and 8 mL of ice-cold, sterile, distilled water. Spores were washed five times by centrifuging at 15,000 x g for 5 min and re-suspending in distilled water. Spores were separated from vegetative material by density gradient centrifugation in Histodenz (Sigma Aldrich, St. Louis, Missouri). Spores were stored at 4°C in sterile distilled water until use. Prior to testing, spore preps were confirmed by phase contrast microscopy and malachite green staining to be >99% dormant, bright-phase spores.

*Bacillus subtilis* spores were prepared at 37°C on 2× SG medium agar plates and harvested, cleaned, and stored as previously described [28]. Spores were separated from vegetative material by density gradient centrifugation in Nycodenz (Axis-Shield, Oslo, Norway). Spores were confirmed by phase contrast microscopy and malachite green staining to be >99% dormant, bright-phase spores.

### Effect of Altered pH on Sporicidal Activity of Alcohol

To determine the effect of altered pH on the sporicidal efficacy of ethanol, the pH of 70% (v/v) ethanol and deionized water was adjusted with hydrochloric acid (HCl) or sodium hydroxide (NaOH) to obtain a range of pHs from 1.3 to >11. Water and ethanol required equal additions of either HCl or NaOH to obtain the desired pH value, therefore the final normalities of water and ethanol solutions were equivalent (i.e. water pH 1.5 = 0.18 N and ethanol pH 1.5 = 0.18 N). The pH measurements were performed using a Fisher Accumet AP71 meter (ThermoFisher Scientific, Waltham, Massachusetts, USA). Ten microliters of spores (~10^6^ CFU) were suspended in one mL of the pH adjusted ethanol or water (baseline), vortexed, and incubated for five minutes at ~22°C (room temperature). *B*. *subtilis*, *B*. *thuringiensis*, and three strains of *C*. *difficile* spores were tested (described above in *Spore Strains and Growth Conditions*). The reaction was quenched by neutralizing 1:1 in Dey-Engley neutralization broth (BD Biosciences, San Jose, California, USA). The efficacy of Dey-Engley neutralization broth was validated for pH 1.3 and >11 aqueous and alcoholic solutions by performing "Standard Test Methods for Evaluation of Inactivators of Antimicrobial Agents" (ASTM 1054–08). Neutralization validation was assessed for all spore strains evaluated in the current study. The test solutions were validated for a one hour hold-time (test solution exposure time), as described in ASTM 1054–08. Neutralized samples were serial diluted in deionized water, drop-plated, and cultured as described above in *Spore Strains and Growth Conditions*. In addition, 1 mL of the neutralized spore suspensions were spread-plated to increase the sensitivity of enumeration for samples with high levels of spore killing. Following incubation, log_10_CFU reduction of spores was determined by calculating the difference in log_10_CFU recovered from baseline (pH altered water) and experimental groups (pH altered ethanol). Similar experiments were conducted to assess whether acidification of 70% (v/v) 1-propanol and 2-propanol to pH 1.5 would result in similar induction of sporicidal activity against *C*. *difficile* spores (strain VA17). In addition, other inorganic and organic acids, including sulfuric, lactic, and citric acids were assessed for their ability to induced sporicidal activity in ethanol against *C*. *difficile* spores (strain VA17).

### Effect of Increased Temperature on Sporicidal Activity of Ethanol Without pH Alteration

Dormant spores are resistant to temperatures up to 80°C for over an hour [20–22, 25]. To determine the effect of elevated temperature on the sporicidal efficacy of ethanol, ten microliters of spores (~10^6^ CFU) were incubated in one mL of 70% ethanol or deionized water at ~22°C (room temperature), 55°C or 80°C. The pH was not altered for these experiments. *B*. *subtilis*, *B*. *thuringiensis*, and VA11 and VA17 *C*. *difficile* spores were tested (described above in *Spore Strains and Growth Conditions*). After 0, 5, 10, 20, 30 and 60 minutes of incubation at the appropriate temperature, 10 μL aliquots of each spore suspension were serial diluted in deionized water, drop-plated, and cultured as described above in *Spore Strains and Growth Conditions*. In addition, 990 μL of the spore suspensions were spread-plated to increase the sensitivity of enumeration for samples with high levels of spore killing. Log_10_CFU reduction of spores was determined by calculating the difference in log_10_CFU recovered from baseline (spores incubated in water) and experimental groups (spores incubated in ethanol).

### Effect of Increased Temperature on Sporicidal Activity of Acidified Ethanol

To assess the effect of increased temperature on sporicidal activity of acidified ethanol, the pH of 70% ethanol and deionized water was adjusted to 1.5 with hydrochloric acid (HCl). Ten microliters of spores (~10^6^ CFU) were inoculated into one mL of the pH adjusted ethanol or water (baseline) and incubated at ~22°C, 55°C or 80°C for five minutes. *B*. *subtilis*, *B*. *thuringiensis*, and three strains of *C*. *difficile* spores were tested (described above in *Spore Strains and Growth Conditions*). The reaction was quenched by neutralizing 1:1 in Dey-Engley neutralization broth (BD Biosciences, San Jose, California, USA). Neutralized samples were serial diluted in deionized water, drop-plated, and cultured as described above in *Spore Strains and Growth Conditions*. In addition, 1 mL of the neutralized spore suspensions were spread-plated to increase the sensitivity of enumeration for samples with high levels of spore killing. Following incubation, log_10_CFU reduction of spores was determined by calculating the difference in log_10_CFU recovered from baseline (pH altered water) and experimental groups (pH altered ethanol).

### Effect of Mild Temperature Elevation and Increased Ionic Strength on Sporicidal Activity of Acidified Ethanol Against *C*. *difficile*

We assessed the effect of increased ionic strength on the sporicidal activity of acidified ethanol against *C*. *difficile* (strain VA17) at room temperature (22°C) and at 42°C, a moderate temperature that is tolerable on skin. The pH of 70% ethanol was adjusted to 3.0, 2.0, 2.5, and 1.5 with hydrochloric acid (HCl). Additionally, the acidified ethanol solutions were buffered with incremental quantities of sodium hydroxide (NaOH), yielding solutions with increasing ionic strength. Ionic strength (*I*) was calculated using the following formula:

I=1/2×[(concentration(molarity)of HCl×#of hydrogen ions×(charge of hydrogen)2)+(concentration of HCl×#of chloride ions×(charge of chloride)2)+(concentration of NaOH×#of sodium ions×(charge of sodium)2)+concentration of NaOH×#of hydroxide ions×(charge of hydroxide)2)]

Ten microliters of spores (~10^6^ CFU) were inoculated into one mL of the pH adjusted ethanol, ethanol (without altered pH), or water (baseline) and incubated in a water bath at 22°C or 42°C for one and ten minutes. The reaction was quenched by neutralizing 1:1 in Dey-Engley neutralization broth (BD Biosciences, San Jose, California, USA). Neutralized samples were serial diluted in deionized water, drop-plated, and cultured. In this experiment, one milliliter of neutralized solution was not spread to increase the sensitivity of detection, therefore the limit of detection was 2 log_10_CFU. Following incubation, log_10_CFU reduction of spores was determined by calculating the difference in log_10_CFU recovered from baseline (pH altered water) and experimental groups (pH altered ethanol). Experiments were performed in triplicate.

### Efficacy of Acidified Ethanol Solutions for Reducing *C*. *difficile* Spores on Hands

A modification of the “Standard Test Method for Determining the Bacteria-Eliminating Effectiveness of Hygienic Handwash and Handrub Agents Using the Fingerpads of Adults” (American Society for Testing and Materials E 2276–10) was used to determine the efficacy of test solutions against non-toxigenic *C*. *difficile* spores [29]. Each fingerpad of both hands were contaminated with 10 μL of a liquid inoculum containing 6 log_10_CFU of ATCC 43593 spores. The fingerpads were rubbed together until the inoculum was dry. Contamination levels were measured using the fingerpad sampling method [29]. In brief, the fingerpads of each hand were rubbed with slight friction against the bottom of a 150 mm x 15 mm Petri dish filled with 25 mL of Dey-Engley neutralizer (BD Biosciences, San Jose, California, USA) for 30 seconds. The neutralizer was collected from the Petri dish, serially diluted 10-fold, and plated on CDBA media to determine *C*. *difficile* counts. Log_10_ reductions were calculated by subtracting log_10_ CFU recovered after hand hygiene treatment from log_10_ CFU recovered from fingerpads without treatment.

A crossover design was used such that each volunteer was exposed to one disinfection procedure or baseline sampling every 24 hours over the span of three days (total of three procedures per volunteer). The order of the hand disinfection procedures for each volunteer was assigned using a computer-generated random numbers list designed to allow all disinfection procedures and one baseline (fingerpads inoculated and then sampled without disinfection) to be tested in triplicate. The person reading the plates to quantify spore counts was blinded to the test product that was used. In initial studies, the hand antiseptic interventions included 1 mL ethanol-based hand sanitizer gel (63% ethanol, Purell, GOJO Industries, Akron, OH), 1 mL of 0.05% triclosan liquid soap (STERIS Corporation, Mentor, OH), 1mL of 10% household bleach solution, and 1 mL of the following acidified ethanol solutions: 70% ethanol (unaltered pH ~5.6), 70% ethanol pH 1.3, 70% ethanol pH 1.5, 70% ethanol pH 2.0, and 70% ethanol pH 2.0 with high ionic strength (buffered with hydrochloric acid and soldium hydroxide). For the soap and water handwash, product was applied directly to the fingerpads only and then rubbed vigorously for 20 sec, rinsed with water until soap was completely removed, and patted dry with paper towels. For the bleach and ethanol-based handrub agents, the agents were applied directly to the fingerpads only and then rubbed together with the agent until they appeared dry.

### Efficacy of Acidified Ethanol for Reducing *C*. *difficile* Spores in an *ex vivo* Porcine Skin Model

To allow an assessment of the efficacy of acidified ethanol in reducing toxin-producing *C*. *difficile* spores on skin, an ex vivo porcine skin model was used. A modified version of ASTM E2897 “Standard Guide for Evaluation of the Effectiveness of Hand Hygiene Topical Antimicrobial Products using *ex vivo* Porcine Skin” was used [30]. In brief, gamma irradiated porcine skin, stored at -80°C, was thawed and cut into 1.5 cm^2^ sections. Ten microliters (~10^6^ CFU) of *C*. *difficile* spores (VA 17 and VA11) were inoculated onto each section, spread to cover the surface of the skin, and incubated at room temperature for 20 minutes to allow the inoculum to dry.

The hand antiseptic formulations included ethanol-based hand sanitizer (Purell, GOJO Industries, Akron, OH), 0.05% triclosan liquid soap (STERIS Corporation, Mentor, Ohio), 10% bleach solution (The Clorox Company, Oakland, California), 70% ethanol adjusted to pH 1.3, 1.5 and 2.0 with HCl, and 70% ethanol adjusted to pH 2.0 with HCl buffered with NaOH (increased ionic strength). To simulate a soap and water hand wash, 50 microliters of soap was pipetted onto an inoculated section of porcine skin and rubbed for 20 seconds with a second inoculated section. Both sections were rinsed with running tap water until soap was removed, and then patted dry on paper towels. For all other formulations and untreated control, 50 microliters of each skin disinfection formulation or water (control) was pipetted onto an inoculated section of porcine skin and rubbed for 30 seconds with a second inoculated section of porcine skin. Following skin disinfection procedures, both sections were placed into a tube containing 10 mL of Dey-Engley neutralizer and vortexed for 2 minutes. Suspensions were serial diluted, drop-plated, and cultured as described above in *Spore Strains and Growth Conditions*. Following incubation, log_10_CFU reduction of spores was determined by calculating the difference in log_10_CFU recovered from baseline (water treated sections) and experimental groups (formulation treated). All skin disinfection formulations were performed in triplicate.

### Data Analysis

*In vitro* data were analyzed using STATA 9.0 (StataCorp, College Station, TX). Continuous data were analyzed using unpaired *t* tests. Error bars indicate standard error. For *ex vivo* skin model experiments, data were analyzed with R statistical software (version 3.1.1). A one-way ANOVA was performed to compare the mean log reductions. A post hoc Tukey HSD test was conducted to test all pairwise differences between group means.The means of the data from experiments conducted are presented.

## Results

### Alteration of pH Induces Sporicidal Activity in Alcohols

**Fig 1:**
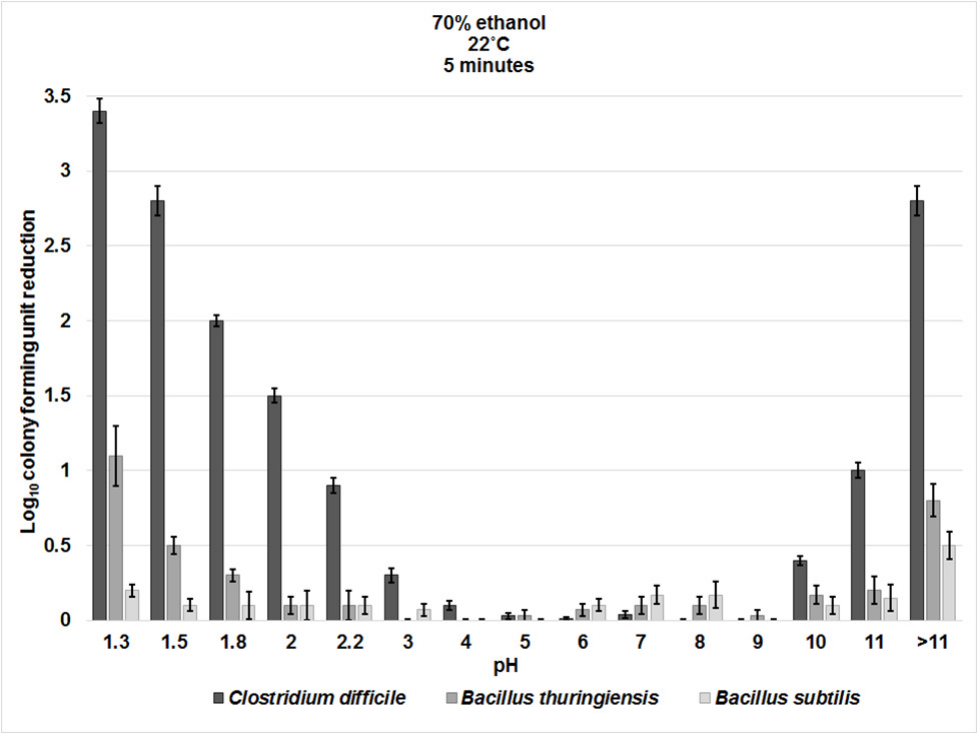
The effect of pH alteration on the spore killing potential of ethanol. Six log_10_colony-forming units (CFU) of *C*. *difficile* (VA17, VA11, and ATCC 43593), *B*. *thuringiensis*, and *B*. *subtilis* spores were exposed to 70% ethanol solutions adjusted to pH 1.3 to >11 for 5 minutes at room temperature. Log_10_CFU reduction of spores was determined by calculating the difference in log_10_CFU recovered from baseline (pH altered water) and experimental groups (pH altered ethanol). The means of data from triplicate experiments are presented. Error bars indicate standard error.

As previously demonstrated, spores remained resistant to pH 1.3 to >11 altered water. There were no significant differences between the log_10_CFU reductions of the three strains of *C*. *difficile* tested (VA11, VA17, and 43593), therefore, data for the strains were pooled (S1 File). *C*. *difficile* spores were reduced by ≥2 log_10_CFU when exposed to 70% ethanol solutions adjusted to pH <2.0 or >11 for 5 minutes at room temperature (Fig 1). *C*. *difficile* spores were significantly more susceptible to killing by acidic and basic ethanol solutions than either of the *Bacillus* spp. (*P*< 0.001 for each comparison); *B*. *thuringiensis* spores were reduced by ~1 log_10_CFU when the pH of ethanol was adjusted to 1.3, whereas no significant reduction of *B*. *subtilis* spores was observed for any of the pH adjusted ethanol solutions.

**Fig 2:**
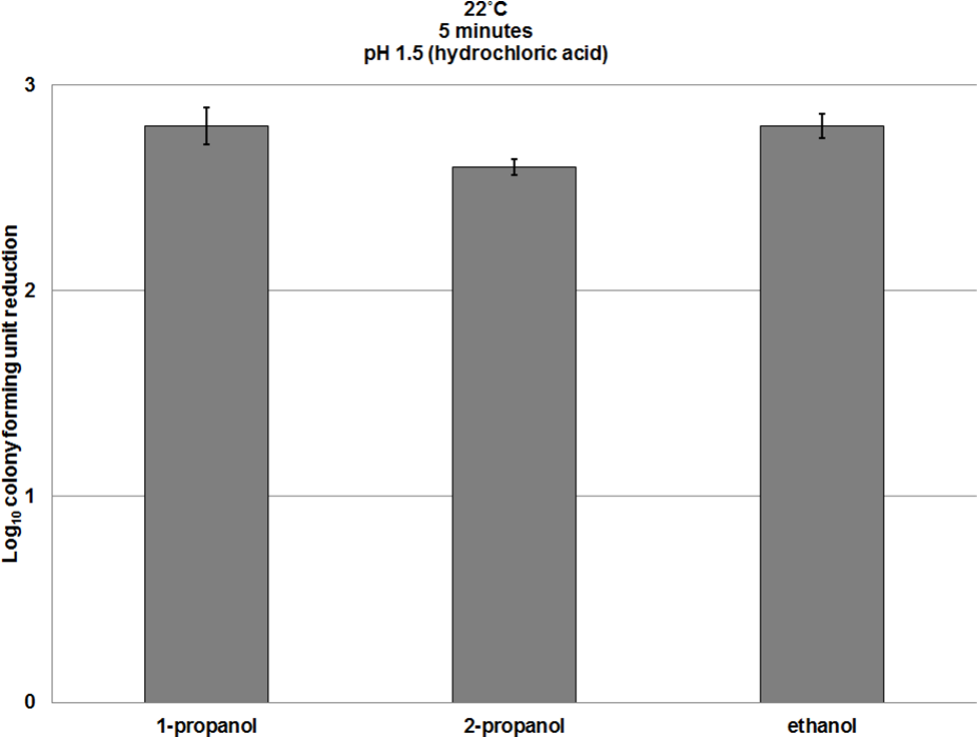
A comparison of the spore killing efficacy of three acidified alcohols. Six log_10_ colony forming units (CFU) of *C*. *difficile* (VA17, VA11, and ATCC 43593) spores were exposed to 70% ethanol, 70% 1-propanol, or 70% 2-propanol solutions adjusted to pH 1.5 for 5 minutes at room temperature. Log_10_CFU reduction of spores was determined by calculating the difference in log_10_CFU recovered from baseline (pH altered water) and experimental groups (pH altered ethanol). The means of the data from experiments conducted in triplicate are presented. Error bars indicate standard error.

**Fig 3:**
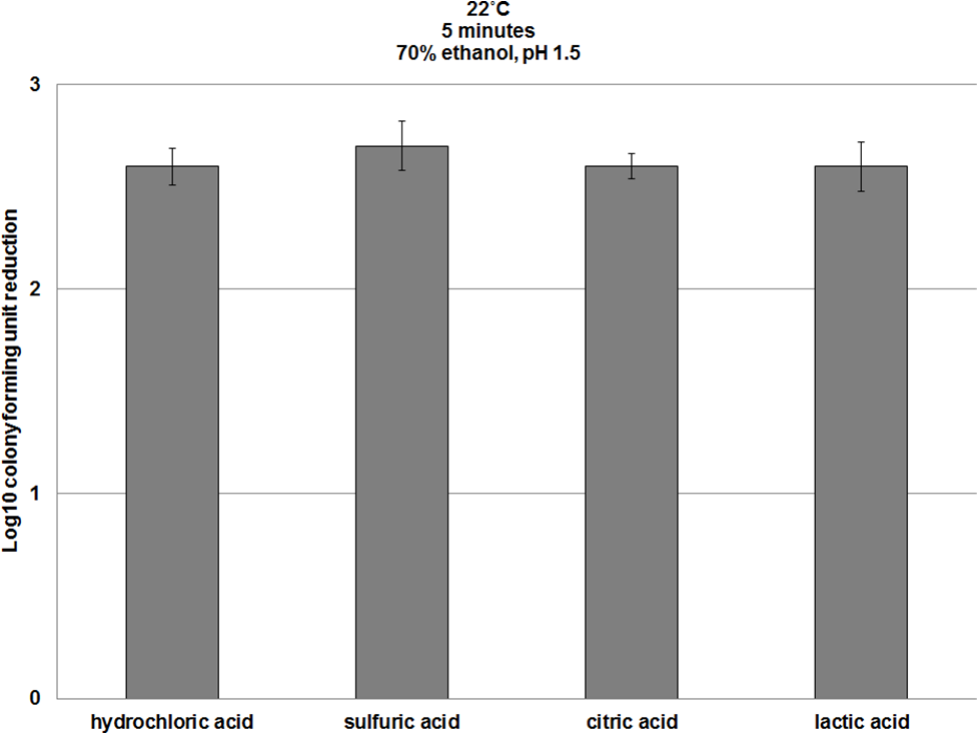
A comparison of the spore killing efficacy of ethanol acidified with organic and inorganic acids. Six log_10_ colony forming units (CFU) of *C*. *difficile* (VA17, VA11, and ATCC 43593) spores were exposed to 70% ethanol adjusted to pH 1.5 with hydrochloric acid, sulfuric acid, citric acid, or lactic acid and incubated for 5 minutes at room temperature. Log_10_CFU reduction of spores was determined by calculating the difference in log_10_CFU recovered from baseline (pH altered water) and experimental groups (pH altered ethanol). The means of the data from experiments conducted in triplicate are presented. Error bars indicate standard error.

The sporicidal effects were not specific to ethanol. Acidification to pH <2 also induced sporicidal activity in 1-propanol (n-propanol) and 2-propanol (isopropanol) against *C*. *difficile* spores (Fig 2, S2 File). In addition, similar results were achieved when the pH was reduced with other inorganic and organic acids, including sulfuric, lactic, and citric acids (Fig 3, S3 File). Based on microscopic appearance, there was no evidence that reductions in spore counts were attributable to spore clumping. Because ethanol is the most common alcohol used for hand sanitizers in the U.S., we focused our remaining experiments on ethanol.

### Elevated Temperature Induces Sporicidal Activity in Ethanol

**Fig 4:**
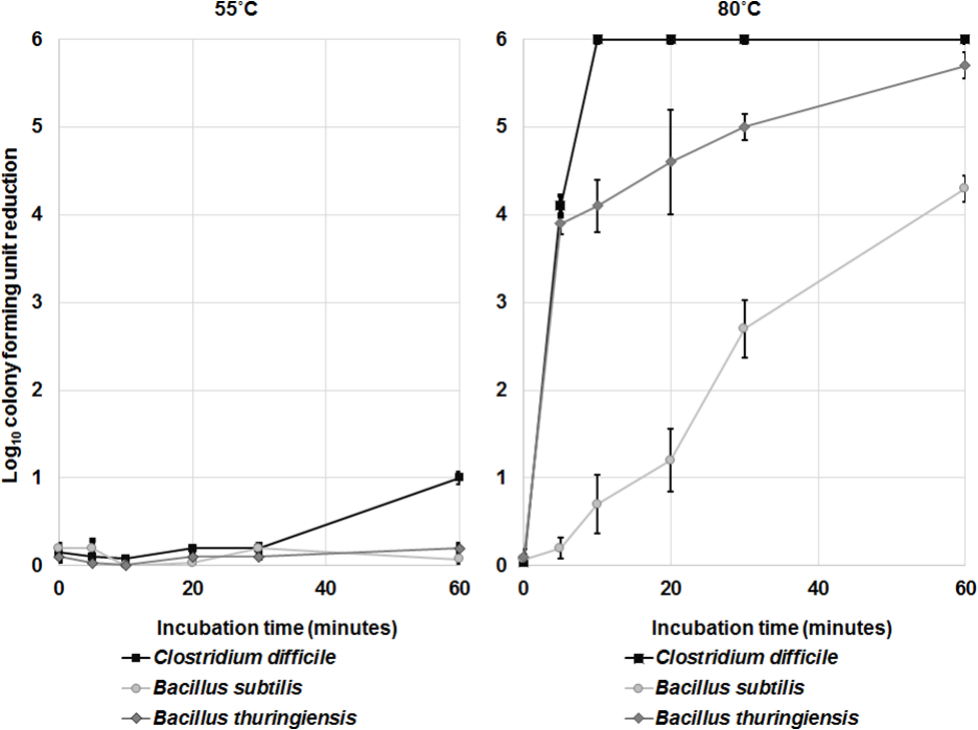
The effect of elevated temperature on the spore killing potential of ethanol. Six log_10_colony-forming units (CFU) of *C*. *difficile* (VA 17 and VA11), *B*. *thuringiensis*, and *B*. *subtilis* spores were exposed to 70% ethanol solutions at 55°C or 80°C for 5, 10, 20, 30 or 60 minutes. Log_10_CFU reduction of spores was determined by calculating the difference in log_10_CFU recovered from baseline (temperature altered water) and experimental groups (temperature altered ethanol). The means of data from triplicate experiments are presented. Error bars indicate standard error.

Increased temperatures of 55°C or 80°C enhance sporicidal activity of antiseptics such as chlorhexidine [20–23]. We therefore examined the effect of these temperatures on sporicidal activity of ethanol with no pH alteration (Fig 4, S4 File). All spores remained 100% viable suspended in water at ~22°C, 55°C, and 80°C for up to 60 minutes (data not shown). However, at 55°C, *C*. *difficile* spores (strains VA11 and VA17) suspended in ethanol were reduced by 1 log_10_CFU after 60 minutes. Killing of spores in ethanol was dramatically increased when the incubation temperature was elevated to 80°C, such that no *C*. *difficile* spores were detectable after 10 minutes of incubation, and after 60 minutes *B*. *thuringiensis* and *B*. *subtilis* were reduced by >5 and >4 log_10_CFU, respectively. These results demonstrate the potential for elevated temperatures to enhance sporicidal activity of ethanol in the absence of pH alteration. However, the temperatures required to achieve sporicidal activity would not be tolerable on skin.

### Mild Temperature Elevation and Increased Ionic Strength Enhance Sporicidal Activity of Acidified Ethanol Against *C*. *difficile*

**Fig 5:**
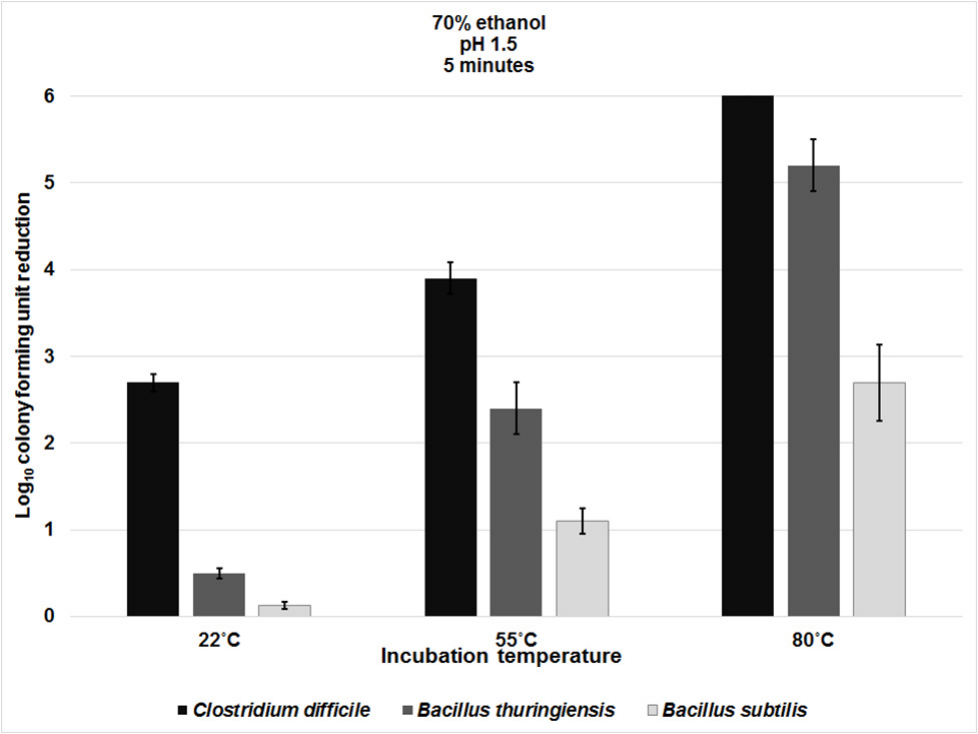
The effect of temperature elevation on the sporicidal activity of acidified ethanol. Six log_10_ colony forming units (CFU) of *C*. *difficile* (VA17, VA11, and ATCC 43593), *B*. *thuringiensis*, and *B*. *subtilis* spores were exposed to 70% ethanol solution adjusted to pH 1.5 and incubated at 22°C, 55°C or 80°C for 5 minutes. Log_10_CFU reduction of spores was determined by calculating the difference in log_10_CFU recovered from baseline (pH altered water) and experimental groups (pH altered ethanol). The means of the data from experiments conducted in triplicate are presented. Error bars indicate standard error.

The sporicidal activity of acidified ethanol pH 1.5 was further enhanced at 55°C and 80°C, with a reduction in *C*. *difficile* spores of ~4 log_10_CFU with a 5 minute exposure, while *B*. *thuriengiensis* were less sensitive to these conditions, and *B*. *subtilis* spores the least sensitive (Fig 5, S5 File). Given the significant sensitivity of *C*. *difficile* spores to acidified ethanol at 55°C, we therefore tested whether milder elevations of temperature that are tolerable on skin (≤42°C) might enhance the sporicidal activity of acidified ethanol. In addition, we tested whether increased ionic strength might further enhance sporicidal activity of acidified ethanol. The rationale for testing solutions with increased ionic strength is that weak ionic bonds in proteins have been shown to be disrupted by solvents containing high ion concentrations (ionic strength), potentially weakening the links in proteinaceous material [31]. Because alcohol hand sanitizers require rapid activity to be effective, we included an exposure time of 1 minute.

**Fig 6:**
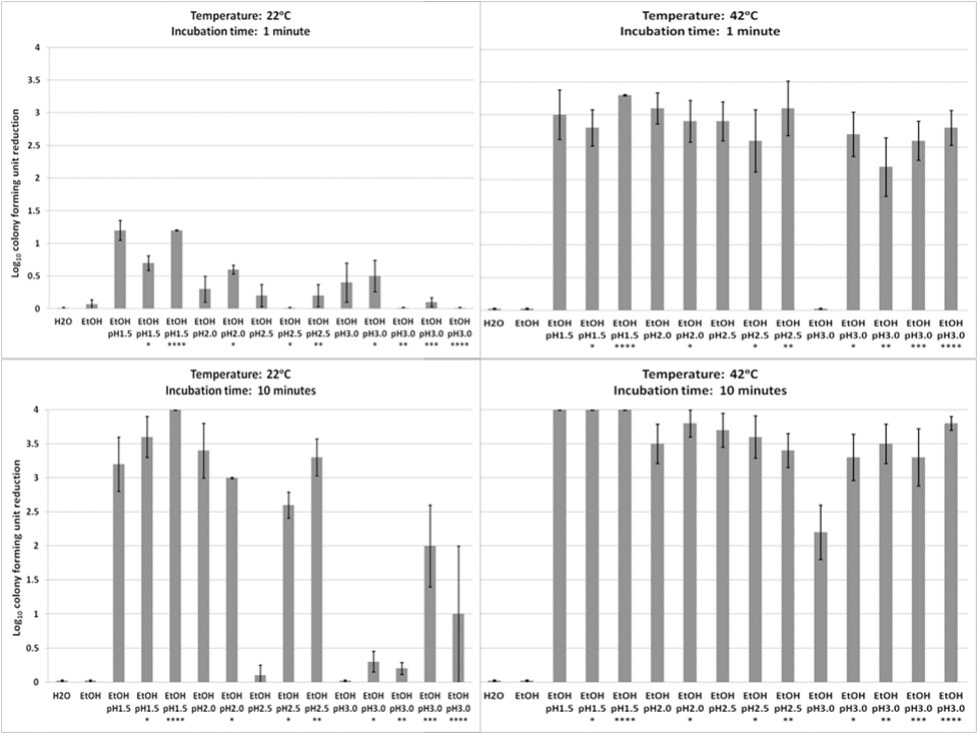
The effect of mild temperature elevation and increased ionic strength on the sporicidal activity of acidified ethanol. Six log_10_ colony forming units (CFU) of *C*. *difficile* (VA17) were exposed to 70% ethanol at room temperature (22°C) and at 42°C for 1 or 10 minutes. Acidified ethanol solutions were adjusted to 3.0, 2.0, 2.5, and 1.5 with hydrochloric acid (HCl). Additionally, the acidified ethanol solutions were buffered with incremental quantities of sodium hydroxide (NaOH), yielding solutions with increasing ionic strength (*I*). Increased ionic strength solutions are labeled “*” to “****” from lowest to highest ionic strength, respectively (I* = 0.2, I** = 0.5, I*** = 0.8, I**** = 1.0). Log_10_CFU reduction of spores was determined by calculating the difference in log_10_CFU recovered from baseline (pH altered water at room temperature) and experimental groups. The means of data from triplicate experiments are presented. Error bars indicate standard error.

Increasing the ionic strength of acidified ethanol solutions and increased temperature of 42°C enhanced sporicidal activity against *C*. *difficile* spores (Fig 6, S6 File). At 22°C, increasing the ionic strength of acidified ethanol solutions significantly enhanced sporicidal activity after 10 minutes of incubation, but no enhancement occurred after 1 minute of incubation. Moreover, a buffered pH 2.5 ethanol solution (*I* = 0.5) performed equivalently to unbuffered pH 1.5 ethanol (*I* = 0.08); both solutions reduced *C*. *difficile* spores by >3 log_10_CFU after 10 minutes of incubation.

At 42°C, the effects of increased ionic strength were masked by the synergistic effect of acidic pH and elevated temperature, with the exception of pH 3.0 solutions. After one minute of incubation at 42°C, spores exposed to pH 3.0 solutions without ionic strength buffering were not killed, whereas pH 3.0 solutions with increased ionic strength reduced *C*. *difficile* spore counts by >2 log_10_CFU for each buffered solution assessed. Similarly, increasing the ionic strength of pH 3.0 solutions enhanced spore killing when incubated for ten minutes at 42°C.

### Acidified Ethanol Reduced Levels of *C*. *difficile* Spores on Skin

**Fig 7:**
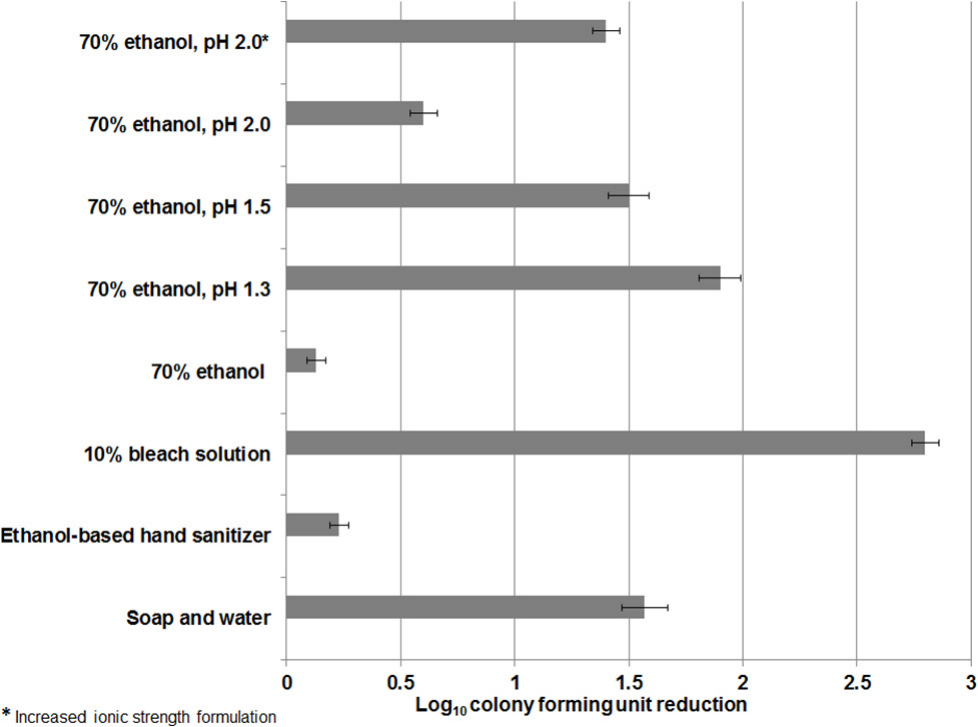
Comparison of soap and water hand wash versus acidified ethanol solutions for removal of non-toxigenic *Clostridium difficile* spores (ATCC 43593) from the finger pads of volunteers. One milliliter of test solution was applied with rubbing to contaminated finger pads. For soap and water hand wash, 1 mL of soap was applied to finger pads, rubbed for 20 seconds, rinsed, and then patted dry with paper towels. Log_10_CFU reduction of spores was determined by calculating the difference in log_10_CFU recovered from treated versus untreated finger pads. The means of data from triplicate experiments are presented. Error bars indicate standard error.

**Fig 8:**
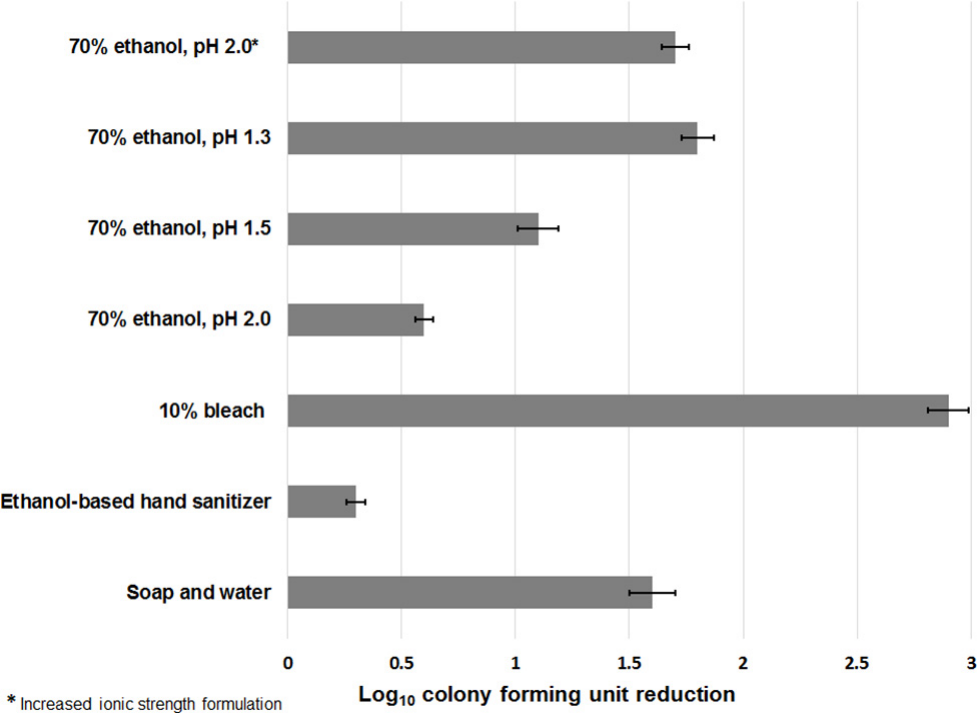
Comparison of soap and water wash versus acidified ethanol solutions for removal of *Clostridium difficile* (VA17 and VA11) spores from porcine skin sections. Fifty microliters of each formulation was pipetted onto an inoculated section of porcine skin and rubbed for 30 seconds with a second inoculated section of porcine skin. To simulate a soap and water hand wash, 50 microliters of soap was pipetted onto an inoculated section of porcine skin and rubbed for 20 seconds with a second inoculated section. Both sections were rinsed with running tap water until soap was removed, and then patted dry on paper towels. Log_10_CFU reduction of spores was determined by calculating the difference in log_10_CFU recovered from treated versus untreated porcine skin sections. The means of the data from experiments conducted in triplicate are presented. Error bars indicate standard error.

Acidified ethanol was effective in reducing recovery of *C*. *difficile* spores (nontoxigenic strain 43593) on hands (Fig 7, S7 File). A soap and water hand wash reduced *C*. *difficile* spores by ~1.5 log_10_CFU, whereas commercial ethanol-based hand sanitizer did not reduce spore counts. Ethanol adjusted to pH 1.5 was as effective as soap and water hand washing in reducing spore recovery from hands. Ethanol adjusted to pH 2.0 with increased ionic strength was also as effective as soap and water (~1.5 log_10_CFU reduction). Similar results were achieved with a toxigenic *C*. *difficile* strain (VA17) using a porcine skin model (Fig 8, S8 File).

## Discussion

Alcohol-based hand sanitizers play a crucial role in protecting healthcare personnel and patients from acquisition of pathogens [1]. However, current hand sanitizer formulations have no activity against bacterial spores produced by pathogens such as *Clostridium difficile* and *Bacillus anthracis* [8–10]. Here, we examined a novel strategy for development of sporicidal sanitizers whereby existing non-sporicidal sanitizers are converted into sporicidal agents through alteration of physical or chemical conditions. We report successful induction of sporicidal activity in ethanol through acidification and further enhancement of activity through increasing ionic strength and mild temperature elevation. Formulations of acidified ethanol were as effective as soap and water washing in reducing levels of *C*. *difficile* spores on skin with a 30 second exposure. These findings suggest that it will be feasible to develop ethanol-based sporicidal hand hygiene products effective for reducing *C*. *difficile* contamination.

Additional studies are needed to identify the mechanism of sporicidal activity of acidified ethanol solutions. We propose that protein denaturation by the acidified ethanol solutions tested here may facilitate rapid penetration of the spore coat, enabling ethanol to reach targets within the spore core. This proposal is consistent with previous demonstrations that conditions that denature proteins may induce sporicidal activity in chlorhexidine and lysozyme [20–23]. Each of the modifications that induced or enhanced sporicidal activity are known to denature proteins. Acids and bases disrupt acidic and basic protein residues [32,33]. High temperatures break hydrogen bonds and hydrophobic interactions in proteins [34]. As noted previously, increased ionic strength may disrupt weak ionic bonds in proteins [31]. Finally, ethanol itself is a protein denaturant that decreases the dielectric constant of water and changes electrostatic interactions in proteins [34]. The potential for protein denaturation to induce sporicidal activity in ethanol is consistent with a recent report that mutation of the spore coat protein *cotA* of *C*. *difficile* results in a major defect in the outer spore coat that induces ethanol susceptibility [35]. To minimize the potential for toxicity to skin, it is likely that an optimal approach for induction of sporicidal activity in ethanol will include a combination of denaturing processes.

*Bacillus* spores, particularly those of *B*. *subtilis*, were more resistant to killing by acidified ethanol solutions than *C*. *difficile* spores. The greater susceptibility of *C*. *difficile* could potentially be due to differences in protein structure of *C*. *difficile* versus *Bacillus* spp. spore coats; recent proteomic studies have revealed major differences in the spore coat and exosporium of *C*. *difficile* and *Bacillus* spp. spores [36]. Differences in spore preparation technique, sporulation medium, and age of spores have previously been shown to effect the thermal resistance of *C*. *difficile* and *Bacillus* spp. spores [37,38]. However, the *C*. *difficile* and *B*. *thuringiensis* spores used in the current study were prepared identically.

Although no adverse effects or discomfort were noted in the volunteers participating in hand hygiene experiments in the current study, safety and tolerability on skin will be important concerns for future development of acidified ethanol formulations because in a clinical setting hand hygiene is performed repeatedly on a daily basis. It is anticipated that acidic solutions with pH 2.5 or below may cause irritation and peeling of skin with repeated exposure. In healthy individuals, the skin surface is mildly acidic (pH 4 to 6) and has been termed an “acid mantle” [39,40]. Mildly acidic skin products in the pH range 3.5 to 4.5 are considered optimal to preserve resident skin microbiota and function [40]. We have demonstrated that pH 2 solutions can be as effective as soap and water hand washing, but additional work is needed to determine if effective formulations can be developed with pH >3.

In summary, we report the development of sporicidal formulations of ethanol. Additional studies are needed to optimize formulations for clinical testing. Further work is also needed to determine if similar strategies will be effective in developing sporicidal formulations of other skin disinfectants such as chlorhexidine.

## Supporting Information

S1 File The effect of pH alteration on the spore killing potential of ethanol. Raw data and statistical analysis for Fig 1.
(XLSX)

S2 File A comparison of the spore killing efficacy of three acidified alcohols. Raw data and statistical analysis for Fig 2.
(XLSX)

S3 File A comparison of the spore killing efficacy of ethanol acidified with organic and inorganic acids. Raw data and statistical analysis for Fig 3.
(XLSX)

S4 File The effect of elevated temperature on the spore killing potential of ethanol. Raw data and statistical analysis for Fig 4.
(XLSX)

S5 File The effect of temperature elevation on the sporicidal activity of acidified ethanol. Raw data and statistical analysis for Fig 5.
(XLSX)

S6 File The effect of mild temperature elevation and increased ionic strength on the sporicidal activity of acidified ethanol. Raw data and statistical analysis for Fig 6.
(XLSX)

S7 File Comparison of soap and water hand wash versus acidified ethanol solutions for removal of non-toxigenic *Clostridium difficile* spores (ATCC 43593) from the finger pads of volunteers. Raw data and statistical analysis for Fig 7.
(XLSX)

S8 File Comparison of soap and water wash versus acidified ethanol solutions for removal of *Clostridium difficile* (VA17 and VA11) spores from porcine skin sections. Raw data and statistical analysis for Fig 8.
(XLSX)
